# Supplementary material for: A Systematic Review and Meta-Analysis of the Effect of Caloric Restriction on Skeletal Muscle Mass in Individuals with, and without, Type 2 Diabetes
Source: Nutrients. 2024 Sep 30;16(19):3328. doi: 10.3390/nu16193328 (PMC11479040; doi:10.3390/nu16193328)

Appendix S1: PRISMA Checklist

Section and Topic	Item #	Checklist item	Location where item is reported
**TITLE**			
Title	1	Identify the report as a systematic review.	Page 1
**ABSTRACT**			
Abstract	2	See the PRISMA 2020 for Abstracts checklist.	Page 2
**INTRODUCTION**			
Rationale	3	Describe the rationale for the review in the context of existing knowledge.	Pages 3 & 4
Objectives	4	Provide an explicit statement of the objective(s) or question(s) the review addresses.	Page 4
**METHODS**			
Eligibility criteria	5	Specify the inclusion and exclusion criteria for the review and how studies were grouped for the syntheses.	Pages 5 & 6
Information sources	6	Specify all databases, registers, websites, organisations, reference lists and other sources searched or consulted to identify studies. Specify the date when each source was last searched or consulted.	Page 5
Search strategy	7	Present the full search strategies for all databases, registers and websites, including any filters and limits used.	Page 5 and Appendices S2-S3
Selection process	8	Specify the methods used to decide whether a study met the inclusion criteria of the review, including how many reviewers screened each record and each report retrieved, whether they worked independently, and if applicable, details of automation tools used in the process.	Page 6
Data collection process	9	Specify the methods used to collect data from reports, including how many reviewers collected data from each report, whether they worked independently, any processes for obtaining or confirming data from study investigators, and if applicable, details of automation tools used in the process.	Page 6
Data items	10a	List and define all outcomes for which data were sought. Specify whether all results that were compatible with each outcome domain in each study were sought (e.g. for all measures, time points, analyses), and if not, the methods used to decide which results to collect.	Page 6
	10b	List and define all other variables for which data were sought (e.g. participant and intervention characteristics, funding sources). Describe any assumptions made about any missing or unclear information.	Page 6
Study risk of bias assessment	11	Specify the methods used to assess risk of bias in the included studies, including details of the tool(s) used, how many reviewers assessed each study and whether they worked independently, and if applicable, details of automation tools used in the process.	Page 7
Effect measures	12	Specify for each outcome the effect measure(s) (e.g. risk ratio, mean difference) used in the synthesis or presentation of results.	Page 7
Synthesis methods	13a	Describe the processes used to decide which studies were eligible for each synthesis (e.g. tabulating the study intervention characteristics and comparing against the planned groups for each synthesis (item #5)).	Pages 7 & 8
	13b	Describe any methods required to prepare the data for presentation or synthesis, such as handling of missing summary statistics, or data conversions.	Page 6
	13c	Describe any methods used to tabulate or visually display results of individual studies and syntheses.	Page 8
	13d	Describe any methods used to synthesize results and provide a rationale for the choice(s). If meta-analysis was performed, describe the model(s), method(s) to identify the presence and extent of statistical heterogeneity, and software package(s) used.	Pages 7 & 8
	13e	Describe any methods used to explore possible causes of heterogeneity among study results (e.g. subgroup analysis, meta-regression).	Page 8
	13f	Describe any sensitivity analyses conducted to assess robustness of the synthesized results.	Pages 7 & 8
Reporting bias assessment	14	Describe any methods used to assess risk of bias due to missing results in a synthesis (arising from reporting biases).	Page 7
Certainty assessment	15	Describe any methods used to assess certainty (or confidence) in the body of evidence for an outcome.	Pages 7 & 8
**RESULTS**			

Section and Topic	Item #	Checklist item	Location where item is reported
Study selection	16a	Describe the results of the search and selection process, from the number of records identified in the search to the number of studies included in the review, ideally using a flow diagram.	Page 9
	16b	Cite studies that might appear to meet the inclusion criteria, but which were excluded, and explain why they were excluded.	N/A
Study characteristics	17	Cite each included study and present its characteristics.	Page 12
Risk of bias in studies	18	Present assessments of risk of bias for each included study.	Pages 18 & 19. Supplementary Figure S1 & Tables S4 & S5
Results of individual studies	19	For all outcomes, present, for each study: (a) summary statistics for each group (where appropriate) and (b) an effect estimate and its precision (e.g. confidence/credible interval), ideally using structured tables or plots.	Pages 13-18
Results of syntheses	20a	For each synthesis, briefly summarise the characteristics and risk of bias among contributing studies.	Pages 13-19
	20b	Present results of all statistical syntheses conducted. If meta-analysis was done, present for each the summary estimate and its precision (e.g. confidence/credible interval) and measures of statistical heterogeneity. If comparing groups, describe the direction of the effect.	Pages 13-18
	20c	Present results of all investigations of possible causes of heterogeneity among study results.	Pages 14-17
	20d	Present results of all sensitivity analyses conducted to assess the robustness of the synthesized results.	Page 18
Reporting biases	21	Present assessments of risk of bias due to missing results (arising from reporting biases) for each synthesis assessed.	Page 19
Certainty of evidence	22	Present assessments of certainty (or confidence) in the body of evidence for each outcome assessed.	Pages 13-18
**DISCUSSION**			
Discussion	23a	Provide a general interpretation of the results in the context of other evidence.	Pages 20-22
	23b	Discuss any limitations of the evidence included in the review.	Page 22
	23c	Discuss any limitations of the review processes used.	Page 22
	23d	Discuss implications of the results for practice, policy, and future research.	Page 22-23
**OTHER INFORMATION**			
Registration and protocol	24a	Provide registration information for the review, including register name and registration number, or state that the review was not registered.	Page 5
	24b	Indicate where the review protocol can be accessed, or state that a protocol was not prepared.	Page 5
	24c	Describe and explain any amendments to information provided at registration or in the protocol.	N/A
Support	25	Describe sources of financial or non-financial support for the review, and the role of the funders or sponsors in the review.	Page 23
Competing interests	26	Declare any competing interests of review authors.	Page 23
Availability of data, code and other materials	27	Report which of the following are publicly available and where they can be found: template data collection forms; data extracted from included studies; data used for all analyses; analytic code; any other materials used in the review.	Page 5

Appendix S2: EMBASE search strategy

Database: Embase <1974 to 2024 May 08>

#	Query	Results from 9 May 2024
1	exp Overweight/	695,875
2	obes*.ti,ab.	586,213
3	overweight.ti,ab.	138,517
4	exp Diabetes Mellitus/	1,263,120
5	diabet*.ti,ab.	1,224,509
6	exp Body Composition/	132,938
7	lean body mass.mp.	12,449
8	muscle mass.mp.	55,211
9	fat free mass.mp.	15,175
10	fat mass.mp.	47,355
11	fat distribution.mp.	14,831
12	exp Caloric Restriction/	19,950
13	low calorie diet.mp.	4,651
14	low energy diet.mp.	917
15	hypocaloric diet.mp.	1,718
16	meal replacement.mp.	864
17	total diet replacement.mp.	104
18	1 or 2 or 3 or 4 or 5	2,057,400
19	6 or 7 or 8 or 9 or 10 or 11	202,871
20	12 or 13 or 14 or 15 or 16 or 17	26,511
21	18 and 19 and 20	3,308
22	exp obesity/	695,875
23	exp diabetic obesity/	4,916
24	exp diabetes mellitus/	1,263,120
25	obes*.mp.	775,976
26	diabet*.mp.	1,523,756
27	overweight.mp.	141,454
28	22 or 23 or 24 or 25 or 26 or 27	2,093,041
29	exp body composition/	132,938
30	body composition.mp.	97,590
31	exp lean body weight/	20,793
32	lean body mass.mp.	12,449
33	muscle mass.mp.	55,211
34	fat free mass.mp.	15,175
35	fat mass.mp.	47,355
36	fat distribution.mp.	14,831
37	29 or 30 or 31 or 32 or 33 or 34 or 35 or 36	216,059
38	exp low calorie diet/	2,602
39	low calorie diet.mp.	4,651
40	low energy diet.mp.	917
41	total diet replacement.mp.	104
42	meal replacement.mp.	864
43	38 or 39 or 40 or 41 or 42	6,253
44	28 and 37 and 43	1,395

Appendix S3: Medline search strategy

Database:
Ovid MEDLINE(R) ALL <1946 to May 08, 2024>

#	Query	Results from 9 May 2024
1	exp Overweight/	282,761
2	obes*.ti,ab.	393,226
3	overweight.ti,ab.	91,662
4	exp Diabetes Mellitus/	526,126
5	diabet*.ti,ab.	805,939
6	exp Body Composition/	65,255
7	lean body mass.mp.	8,965
8	muscle mass.mp.	27,568
9	fat free mass.mp.	9,881
10	fat mass.mp.	27,711
11	fat distribution.mp.	8,722
12	exp Caloric Restriction/	7,366
13	low calorie diet.mp.	1,805
14	low energy diet.mp.	716
15	hypocaloric diet.mp.	1,169
16	meal replacement.mp.	545
17	total diet replacement.mp.	69
18	1 or 2 or 3 or 4 or 5	1,218,085
19	6 or 7 or 8 or 9 or 10 or 11	111,664
20	12 or 13 or 14 or 15 or 16 or 17	10,680
21	18 and 19 and 20	1,336

Table S1: Sensitivity analysis – results from studies reporting full data set only

**T2D analysis**		**Weighted Mean Difference**	**95% confidence interval**	**p-value**
**MM v FM comparison**	**MM**	-2.89kg	-3.55 to -2.24	<0.0001
	**FM**	-7.92kg	-9.92 to -5.91	<0.0001
**MM v BW comparison**	**MM**	-2.84kg	-3.48 to -2.21	<0.0001
	**BW**	-11.27kg	-12.38 to -10.15	<0.0001

**NDM analysis**		**Weighted Mean Difference**	**95% confidence interval**	**p-value**
**MM v FM comparison**	**MM**	-2.80kg	-3.36 to -2.24	<0.0001
	**FM**	-6.97kg	-7.61 to -6.33	<0.0001
**MM v BW comparison**	**MM**	-2.76kg	-3.32 to -2.20	<0.0001
	**BW**	-9.89kg	-10.67 to -9.11	<0.0001

Table S2: Sensitivity analysis – result by measure of MM

	**Number of cohorts**	**Weighted Mean Difference**	**95% confidence interval**	**p-value**
**FFM**	36	-2.80kg	-3.27 to -2.33	<0.0001
**LBM**	12	-3.03kg	-3.94 to -2.12	<0.0001
**SMM**	3	-1.69kg	-3.24 to -0.14	0.03
	**Test of group differences**			**0.33**

Table S3: Sensitivity analysis – result by method of body composition assessment

	**Number of cohorts**	**Weighted Mean Difference**	**95% confidence interval**	**p-value**
**Air displacement plethysmography**	7	-2.06kg	-3.10 to -1.02	<0.0001
**Bioimpedance analysis**	15	-2.50kg	-3.56 to -1.43	<0.0001
**Dual-energy X-ray absorptiometry**	10	-3.11kg	-3.72 to -2.49	<0.0001
**Deuterium dilution**	12	-2.18kg	-3.19 to -1.18	<0.0001
**Underwater weighing**	3	-3.98kg	-5.54 to -2.43	<0.0001
	**Test of group differences**			**0.32**

Figure S1: Risk of bias assessment results for RCT studies

	Risk of bias domains					
**Study**	**D1**	**D2**	**D3**	**D4**	**D5**	**Overall**
Rolland *et al*., 2011	-	+	+	+	+	-
Vink *et al*., 2016	!	+	+	+	+	!
Christensen *et al*. 2005	!	+	+	+	+	!
Ng Tang Fui *et al*., 2016	+	+	+	+	+	+
Liljensøe *et al*., 2021	+	!	+	+	+	!
Brown *et al*., 2020	+	+	+	+	+	+
Behary *et al*., 2019	+	+	+	+	+	+
Vink *et al*., 2017	!	!	+	+	+	!
Soenen *et al*., 2013	!	+	+	+	+	!
Munro & Garg, 2013	+	+	+	+	+	+

Table S4: Quality assessments for single group interventional studies

	Borg et al., 2002	Bucci et al., 2015	Westerterp-Plantenga et al., 2004	Carella et al., 1997	Vogels et al., 2007	Nymo et al., 2018	Munro & Garg., 2011	Tomlinson et al., 2004	Hursel & Westerterp, 2009	Claessens et al., 2009	Uusi-Rasi et al., 2010	Lundgren et al., 2021	Iepsen et al., 2016
**Selection**													
**1. Representativeness of the exposed cohort**	a	a	a	a	a	a	a	a	a	a	a	a	a
**2. Selection of the non exposed cohort**													
**3. Ascertainment of exposure**	a	a	a	a	a	a	a	a	a	a	a	a	a
**4. Demonstration that outcome of interest was not present at start of study**	a	a	a	a	a	a	a	a	a	a	a	a	a
**Comparability**													
**1. Comparability of cohorts on the basis of the design or analysis**													
**Outcome**													
**1. Assessment of outcome**	a	a	a	a	a	a	a	a	a	a	a	a	a
**2. Was follow-up long enough for outcomes to occur**	a	a	a	a	a	a	a	a	a	a	a	a	a
**3. Adequacy of follow up of cohorts**	a	b	b	b	b	b	b	a	b	b	b	b	a
**Number of stars**	**6**	**6**	**6**	**6**	**6**	**6**	**6**	**6**	**6**	**6**	**6**	**6**	**6**

	Luscombe et al., 2006	Martins et al., 2023	Camps et al., 2019	Sayda et al., 2022	Vogels & Westerterp, 2005	Belza et al., 2009	Näätänen et al., 2023	Adam et al., 2006	Näätänen et al., 2021	Schulte et al., 2012	Packianathan et al., 2005	Krotkiewski et al., 1990	Hoie et al., 1993
**Selection**													
**1. Representativeness of the exposed cohort**	a	a	a	a	a	a	a	a	a	a	a	a	a
**2. Selection of the non exposed cohort**													
**3. Ascertainment of exposure**	a	a	a	a	a	a	a	a	a	a	a	a	a
**4. Demonstration that outcome of interest was not present at start of study**	a	a	a	a	a	a	a	a	a	a	a	a	a
**Comparability**													
**1. Comparability of cohorts on the basis of the design or analysis**													
**Outcome**													
**1. Assessment of outcome**	a	a	a	a	a	a	a	a	a	a	a	a	a
**2. Was follow-up long enough for outcomes to occur**	a	a	a	a	a	a	a	a	a	a	a	a	a
**3. Adequacy of follow up of cohorts**	b	a	a	a	a	b	b	c	a	a	b	b	b
**Number of stars**	**6**	**6**	**6**	**6**	**6**	**6**	**6**	**5**	**6**	**6**	**6**	**6**	**6**

	Scragg et al., 2020	Jian et al., 2022	Diepviens et al., 2007	Christensen et al., 2018	Marples et al., 2022	Lim et al., 2011
**Selection**						
**1. Representativeness of the exposed cohort**	b	b	a	b	b	b
**2. Selection of the non exposed cohort**						
**3. Ascertainment of exposure**	a	a	a	a	a	a
**4. Demonstration that outcome of interest was not present at start of study**	a	a	a	a	a	a
**Comparability**						
**1. Comparability of cohorts on the basis of the design or analysis**						
**Outcome**						
**1. Assessment of outcome**	a	a	a	a	a	a
**2. Was follow-up long enough for outcomes to occur**	a	a	a	a	a	a
**3. Adequacy of follow up of cohorts**	b	a	a	b	c	b
**Number of stars**	**6**	**6**	**6**	**6**	**5**	**6**

Table S5: Quality assessments for multiple-group non-RCT studies

	Tam et al., 2016	Aukan et al., 2023	Athithan et al., 2023	van Dale et al., 1990a	Ivan et al., 2022	Nordstrand et al., 2013	van Dale et al., 1990b
**Selection**							
**1. Representativeness of the exposed cohort**	b	b	a	a	a	b	a
**2. Selection of the non exposed cohort**	a	a	a	a	a	a	a
**3. Ascertainment of exposure**	a	a	a	a	a	a	a
**4. Demonstration that outcome of interest was not present at start of study**	a	a	a	a	a	a	a
**Comparability**							
**1. Comparability of cohorts on the basis of the design or analysis**	a	a	a	a	a	a	a
**Outcome**							
**1. Assessment of outcome**	a	a	a	a	a	a	a
**2. Was follow-up long enough for outcomes to occur**	a	a	a	a	a	a	a
**3. Adequacy of follow up of cohorts**	b	b	b	a	a	b	b
**Number of stars**	**8**	**8**	**8**	**8**	**8**	**8**	**8**

Figure S2: Funnel plot of studies included in NDM analysis

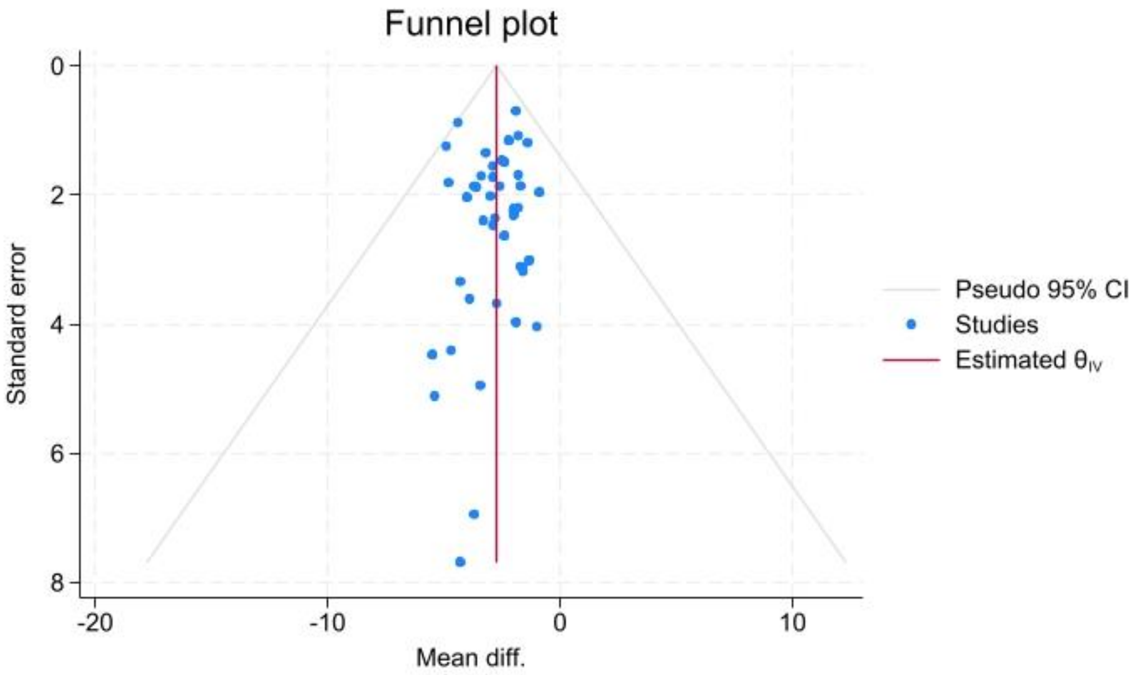

Supplement: Supplementary file 1 [file nutrients-16-03328-s001.zip › nutrients-3199811-supplementary.pdf]
